# Moral lessons from residents, close relatives and volunteers about the COVID-19 restrictions in Dutch and Flemish nursing homes

**DOI:** 10.1186/s13010-023-00140-w

**Published:** 2023-09-06

**Authors:** Elleke Landeweer, Nina Hovenga, Suzie Noten, Floor Vinckers, Jasper de Witte, Annerieke Stoop, Sytse Zuidema

**Affiliations:** 1 Department of Primary and Long-term care, University of Groningen, University Medical Center Groningen, Groningen, 9713 GZ The Netherlands; 2 TRANZO, Tilburg School of Social and Behavioral Sciences, Tilburg University, P.O. Box 90153, Tilburg, 5000 LE The Netherlands; 3 HIVA- Research Institute for Work and Society, KU Leuven, P.O. Box 5300, 3000 Leuven, Belgium

**Keywords:** COVID-19, Nursing homes, Restrictive measures, Moral lessons, Residents, Close relatives, Volunteers

## Abstract

**Background:**

During the COVID-19 outbreak in 2020, national governments took restrictive measures, such as a visitors ban, prohibition of group activities and quarantine, to protect nursing home residents against infections. As ‘safety’ prevailed, residents and close relatives had no choice but to accept the restrictions. Their perspectives are relevant because the policies had a major impact on them, but they were excluded from the policy decisions. In this study we looked into the moral attitudes of residents, close relatives and volunteers regarding the restrictions in retrospect, and what moral lessons they considered important.

**Methods:**

We conducted 30 semi-structured interviews with residents and close relatives and one focus group meeting with volunteers working in nursing homes. Data were transcribed verbatim and analyzed inductively. Subsequently, three Socratic dialogue meetings with residents, close relatives and volunteers were organized in which first analysis outcomes were discussed and dialogues were fostered into moral lessons for future pandemics. Outcomes were combined with moral theory following an empirical bioethics design.

**Results:**

Critical perspectives regarding the COVID-19 restrictions grew in time. Various moral values were compromised and steered moral lessons for our future. The participants recognized three moral lessons as most important. First, constructing tailored (well-balanced) solutions in practice is desirable. Second, proper recognition is needed for the caring role that close relatives fulfill in practice. Third, a responsive power distribution should be in place that includes all stakeholder perspectives who are affected by the restrictions.

**Discussion:**

Comparing the results with moral theory strengthens the plea for inclusion of all stakeholder groups in decision-making processes. To further concretize the moral lessons, tailored solutions can be realized with the use of moral case deliberations. Proper recognition includes actions addressing moral repair and including counter-stories in the debate. Responsive power distribution starts with providing clear and trustworthy information and including all perspectives.

**Supplementary Information:**

The online version contains supplementary material available at 10.1186/s13010-023-00140-w.

## Background

When the COVID-19 pandemic hit the Netherlands and Flanders (Belgium), the national governments obliged nursing homes (nursing homes) to prohibit in-person visits. This implied that close relatives and most volunteers were no longer allowed to visit residents in nursing homes. In addition, communal spaces closed and all group gatherings and social activities were cancelled [1, 2]. Moreover, in several elderly care organizations, residents were obliged to stay in quarantine in their assigned rooms. The purpose of these restrictions was to prevent the virus from spreading and infecting residents and staff in the nursing homes (i.e. to prevent severe disease and deaths) [3]. The consequence of these restrictions was that residents became physically isolated from their close relatives for several months at the risk of increasing loneliness and decreasing quality of life and wellbeing. Most of the residents stayed in contact with their relatives from a distance, for instance by (video) calling each other or waving at each other through windows [4]. As it was known that close relatives have a positive influence on the wellbeing of residents [5, 6], it was expected that these restrictions risked negatively affecting the lives of residents.

Soon after the restrictions were implemented, various newspapers reported stories on how the ban on in-person visits affected residents, describing an increase in loneliness and reduced quality of life. With that, it was questioned if the restrictions were worth the costs [7–9]. In addition, scientific studies started to publish about the (potential) harm of social isolation affecting the mental wellbeing of residents [10–14]. Research also showed that the measures did not fully prevent against a rapid spread of the virus in nursing home [15]. At the same time, studies into the (normative) perspectives and lived experiences of the people whose lives were most affected (i.e. residents, close relatives and volunteers) were not conducted despite the relevance of their perspectives given that the policies significantly impacted them [16]. Therefore, we studied the impact of the restrictive COVID-19 measures on the lives of residents, close relatives and volunteers regarding the fulfillment of their social needs, effects on loneliness and what lessons could be learned within a larger multicenter qualitative research project [17]. As part of this project we looked at how residents, close relatives and volunteers, in retrospect, judged the restrictions. We asked them which values and norms they considered (most) important and what kind of normative lessons should be considered in the future. We were particularly interested in their opinions as they were scarcely involved in the decision-making process about the restrictive measures that were taken. In this article, we present their normative outlooks and lessons, and discuss the meaning and relevance for future policies.

## Methods

The present study was conducted within a larger research project on the consequences of COVID-19 outbreak restrictive measures, such as loneliness, social needs and resilience on experiences of nursing home residents, families and volunteers. This project was funded by The Netherlands Organization for Health Research and Development (grant number ZonMw 10430022010010). The research project addressed the following research questions: 1)What is the impact of restrictive measures on experiences of loneliness and social needs of nursing home residents, close relatives and volunteers?; 2) What is the impact of restrictive measures on social relationships and social contact of nursing home residents, families and volunteers?; 3) What is the impact of restrictive measures on the resilience of nursing home residents, close relatives and volunteers and how they help to diminish consequences of restrictive measures?; 4) What are lessons learned for policy and healthcare delivery in case of a second wave outbreak of COVID-19 to diminish consequences for nursing home residents, families and volunteers? This research project was carried out by three academic networks in which universities collaborate with regional healthcare organizations for older adults (i.e. UNO-UMCG, University of Groningen; Tranzo, Tilburg University; HIVA, KU Leuven). As data collection was performed in different regions in the Netherlands and in the Dutch-speaking part of Belgium with different measures and severity of COVID-19 infections, we were able to include a diverse group and gather knowledge about different contexts. We aimed to gain a deep understanding of the impact of the measures for residents, relatives and volunteers. We decided to take all results together as no large differences between the regions were observed.

Based on the research outcomes of the project, three different overarching themes were distinguished: (1) fulfillment of social needs and related negative emotions, including loneliness [17]; (2) resilience and resources to alleviate the negative impact of restrictive measures; and (3) moral attitudes and moral lessons from the perspective of residents, close relatives and volunteers.

### Study design

 The present qualitative study focused on the moral attitudes and moral lessons of residents, relatives and volunteers. For this qualitative study, we used the data of ***30 interviews*** conducted with nursing home residents and close relatives (either the resident together with their relative or separate) and ***one focus group*** with volunteers. We analyzed the data for moral context and identified the (underlying) values and moral issues experienced by nursing home residents, close relatives and volunteers. In addition, we fostered a dialogue on moral stances and moral issues in three ***Socratic dialogues meetings***. Socratic dialogue is a method to foster philosophical conversations with a group of participants. This method invites participants to reflect critically on topics through dialogue. Traditionally the central aim of Socratic dialogue is to find truth. Nowadays Socratic dialogue aims to stimulate critical thinking and develop practical wisdom. Elements of this method contained in this study included: (1) a concrete topic which in this study was experienced values and norms concerning the visitors ban; (2) pinpointing crucial moments (examples) where values and norms were compromised; (3) mutual analysis of the essence of those experiences; and (4) reflection about if or what conclusions could be possible (in this study formulating moral lessons) [18].

In this study, the Socratic dialogue meetings consisted of a mix of stakeholder perspectives representing nursing home residents, close relatives and volunteers. The aim was to encourage mutual analysis to develop deeper insights into normative interpretations of the contextual backgrounds that steered the participants’ experience [19]. The method of Socratic dialogue was relevant to answer the research question as participants were strongly invited to support their moral intuitions with concrete situations they had experienced and to compare their moral intuitions with each other to reflect on their positions. Central questions were: ‘What should not be forgotten in case a new pandemic would develop?’ and ‘What are minimal criteria that should be met?’ The role and position of the facilitator was that of a Socratic guide. The first two authors facilitated these meetings, as they were both trained as facilitators in moral case deliberation and experienced in using the method of Socratic dialogue [20, 21].

### Recruitment and data collection

Participants were affiliated with psychogeriatric and somatic wards in Flanders (Belgium) and in the Northern and Southern regions of the Netherlands. Recruitment took place via nursing homes as well as social media. The project team approached nursing homes in the summer of 2020. At this time, the visitors ban had stopped but there were still some restrictions such as wearing a face mask and keeping distance in public areas. Care professionals were asked to identify eligible participants and provided them an information letter explaining the purpose of the study. In case a resident had reduced capacity due to dementia, their legal representative was also informed about the study. In addition, an announcement was placed on the website and LinkedIn-page of the research departments, which resulted in one response. Inclusion criteria were that 1) residents had to live in a nursing home in the northern, eastern or southern regions of the Netherlands, or in Flanders, Belgium at the time of the visitors ban in March 2020. Close relatives were defined as individuals who had a relative or partner living in a nursing home during that period. Volunteers were defined as persons who work for nursing homes without being paid. Volunteers had to be affiliated with a nursing home during the visitors ban. Participants had to be able to verbally communicate in Dutch. Various background variables were considered, such as sex, gender, severity of physical and cognitive conditions, type of relatives (i.e. child, sibling, partner), different types of volunteers (i.e. age, duration of involvement in the nursing home, type of voluntary work and severity level of COVID-19 outbreak).

All participants were informed about the research and provided written consent for recording and consented to the publication of their anonymized data. In cases of residents with reduced cognitive capacities (i.e. dementia), proxy consent was obtained. Residents with severe dementia were not included.

Interviews were conducted face-to-face in residents’ nursing homes and were intended to last not longer than 60 min. During data collection, researchers wore face masks and respected the social distancing rules that applied at that time. The focus group with volunteers and the Socratic dialogue meetings took place online and took around 90 min. Guides used for the interviews, focus groups and Socratic dialogue meetings are added as an Appendix.

Participants of the interviews varied in age (57–101 year), role (residents/ close relatives) as well as between regions (Northern Netherlands, Southern Netherlands, Flanders). All volunteers participating in the focus group meeting worked in nursing homes in the various regions before the outbreak of the COVID-19 pandemic (N = 10). Two volunteers stayed involved during the restrictions in the nursing homes, others were forced to stop or decided to stop their voluntary work in the nursing homes. The three organized Socratic dialogue meetings consisted of a mixed group of participants. In total two residents, five close relatives and six volunteers joined these meetings.

### Data analysis

The interviews and focus group meetings were audio recorded and transcribed verbatim. Within the research group, all transcripts were thematically analyzed and coded with the use of Atlas.ti version 8 software. An open, inductive approach was used for analysis. First, researchers (EL, NH, FV, JW, AS and SN) read and discussed the transcript of the first interview. Next, EL, FV, JW and SN coded the transcript of the interview independently and compared the results. Discussion led to a first set of codes. The same steps were performed on the second interview and a code tree was constructed. The rest of the interviews were divided among the researchers per region. A researcher from another region performed a check on the coding to increase the inter-researcher reliability. Differences were discussed during meetings and all researchers agreed on the final coding (e.g. negative case analysis, peer debriefing). The participants received a member check to approve the content and to provide an opportunity to give additional suggestions to their given information. This resulted in a few minor details that were added.

After the coding, we specifically searched for codes that presented moral emotions and normative stances, inspired by the methodology of empirical bioethics [22, 23] using interpretive elements inspired by the hermeneutic philosophy of Gadamer [24, 25] to develop a deep understanding of moral attitudes and values and norms involved.

In addition, the Socratic dialogue meetings were analyzed iteratively using interpretative phenomenological analysis [26] on how participants experienced the other’s point of view and mutual consensus was developed in dialogue. All researchers first analyzed the transcripts deductively by coding expressed values and norms. These codes were grouped and values and norms clustered where possible. In the next phase, three researchers (EL, NH, FV) reread the transcripts, inductively looking at how the codes developed during the dialogue to interpret how the normative position of the codes developed in dialogue. Specific attention was given to the critical normative perspectives, as these were most informative about which values were experienced as morally at stake or compromised and how lessons were constructed.

## Results

Thirty interviews were conducted: nine in the northern and eastern part of the Netherlands, eight interviews in the southern Netherlands, and thirteen in Flanders. Nineteen interviews consisted of residents and close relatives together, and 11 interviews took place individually (seven with residents and four with close relatives). Residents were living at psychogeriatric, somatic, or combined units of nursing homes and ranged in age from 57 to 101 years (7 males and 23 females). Eleven residents had been infected by the COVID-19 virus. Close relatives were 12 daughters, seven partners, two sons, a daughter-in-law, and a brother. Ten volunteers (aged 59 to 76 years, 2 males and 8 females, with 2 to 14 years of volunteer work in nursing homes) participated in the focus group. In the Socratic dialogue meetings, 5 close relatives ( 3 partners, 1 daughter in law, 1 daughter), two residents and six volunteers participated.

First, we present the moral perspective of how the participants experienced the restrictions in nursing homes and to what extent the restrictions were critically judged. Second, we zoom in to consider which values and norms were experienced as most important to the participants and why. Third, we present normative lessons formulated by the participants based on their experiences with the restrictions.

### Moral attitudes regarding the restrictions in nursing homes

In retrospect, most participants were nuanced in their moral judgments about the restrictive COVID-19 measures in nursing homes. Although they reported the in-person visit ban as challenging (i.e. experiencing various negative emotions such as worries, frustration, fear, boredom and loneliness), they did not explicitly judge the restrictive measures during the first period of the COVID-19 outbreak as morally wrong. They reported that they initially accepted the restrictions because nobody knew what to expect of the outbreak. When time passed, several participants mentioned they started to feel less at ease with the restrictions and developed critical moral perspectives.

“It was…everyone is willing to make a sacrifice, my mother as well. So at the beginning it was all going pretty well. But at a certain point that ends. For us too, we were like ‘oh, come on’!” (GH3).

#### Critical perspectives

Although most participants also accepted the restrictions in retrospect, explicit critical standpoints regarding the in-person visit ban were expressed. These critical perspectives are informative to learn which values were experienced as morally under pressure. Below we distinguish the critical outlooks in various themes and related argumentations, i.e. the restrictions were disproportional, not fair, and/or not rightfully imposed.

##### Restrictions experienced as disproportional

Some participants argued that the restrictions did not protect the residents sufficiently, as the virus still spread in the nursing homes. Therefore, they concluded that the protection argument used to justify the ban of in-person visits was (in retrospect) invalid. In addition, participants reported that various regulations were experienced as inconsistent or illogical. An example of inconsistency was that residents were still allowed to join group gatherings in churches while in-person visits were not allowed. Another example was a close relative observing from outside how a care professional comforted her loved one by putting an arm around him, not keeping a distance.

Participants of the Socratic dialogue meetings searched for a well-balanced equilibrium between the pros and cons related to the restrictions. They weighed for example the negative consequences for residents and their close relatives (i.e. loneliness, anxiety) against the motives for the restrictions (i.e. safety/ protection). Strong metaphors were used to emphasize the impact of the restrictions, referring to being in prison or war. In retrospect, participants in the Socratic dialogue meetings concluded that the price residents had to pay for staying safe might have been too high because safety was still not guaranteed.

“In a phone call a resident said “someone in prison is better off than us, because than you are allowed to walk outside for an hour at the courtyard, while we aren’t even allowed to leave our room”. (focus group with volunteers R8)

##### Restrictions experienced as not fair

A second critical perspective referred to the question of who should be allowed to continue care activities in nursing homes. While policies varied between nursing homes if volunteers were allowed to continue their volunteer work during the lockdown, volunteers who continued their work struggled with the fact that they were allowed to enter the nursing home, while close relatives were not. On the one hand, it was considered as beneficial for the residents that -at least- volunteers were still allowed to contribute to residents’ wellbeing. On the other hand, volunteers felt uncomfortable and guilty towards close relatives as it was experienced as unfair that they were allowed to continue their work. The elderly care organization of one of the participants stopped with its volunteer work for that reason:

“On a certain moment they said: now we also put the volunteers on hold for a while, because we can’t justify the visitors-ban for family members, while allowing a volunteer to be with the resident.” (Focus group with volunteers R6).

Residents and close relatives reported they felt discriminated against for not having a voice in the matter. They reported feeling powerless. In addition, there were close relatives who felt wrongly identified a visitor, as they used to be closely involved in caring for their loved one. One partner experienced it as extremely humiliating that he, as partner, was reduced to the role of visitor while he conducted more caring tasks for his wife who was living in the nursing home than the care professionals who were working there. Also, because he considered himself as less of a risk to get infected by the virus than care professionals, he experienced it as unfair that he was not allowed to care for his wife.

“Yes, well, how shall I put it. I didn’t agree, that’s for sure. I was assisting her every day, with all of the caring and what so ever, and then all of a sudden you’re being disposed of as if you’re a danger. While I think, if they had let me in during this period, there would have been less risk of infection, because during the day about six or more caretakers come by here on a regular basis and if I would have been here on my own, in my opinion the risk would be six times less”. (T5)

##### Restrictions experienced as not rightfully imposed

A third critical perspective focused on how restrictions were operationalized within elderly care organizations. Participants noticed variations between nursing homes in how the restrictions were implemented. In some nursing homes, residents had to stay in their apartments/rooms, while in others they were still allowed to go outside. Also, there was variation in how nursing homes communicated about the restrictions. Residents and families reported having missed clear information and communication from their nursing homes frequently, for example about what was and was not allowed. While the regulations changed regularly over a certain period, not all close relatives knew which rules they were obliged to follow. In addition, in general close relatives reported that it was hard to get in touch with their organization and that they received neither information about the restrictions nor how their loved one in the nursing home was doing. This increased their anxiety and frustration. They missed consideration and recognition for their position. These differences appealed to feelings of injustice because the nursing home you were affiliated with determined how you were treated.

### Values and norms recognized as important

In the interviews and focus groups, participants were asked which values were compromised due to the restrictions. In addition, the argumentations of the participants’ critical perspectives showed what was considered of value and morally at stake, which steered the debate in the Socratic dialogue meetings to how these values could be meaningful in daily practice (i.e. what kind of *implicit* rules, routines and behaviors would be appropriate). In other words, if they connected any norms to these values.

#### Safety

All participants recognized safety and protection of residents as highly valuable, but varied in perspectives about how this should be operationalized in daily practice. It was reckoned that within the scope of safety in daily practice close relatives and volunteers who have limited social contacts in their personal lives, should be allowed in nursing homes. Their COVID-19 transmission risk was considered lower than care professionals, who often have a family life with multiple housemates. In other words, relatives were not jeopardizing safety as much as care professionals.

#### Mental well-being

Besides safety, close relatives and volunteers often mentioned the mental wellbeing of the residents as an important aspect that should also be taken into account. In this context, it was emphasized that staying in touch with the residents is a norm that close relatives and volunteers should address. The social and physical presence of loved ones could make a major difference for some residents, such as preventing feelings of loneliness. One of the volunteers described how she experienced not being allowed to visit the resident she looked after in the nursing home. She believed it must have been very lonely for this resident also because video chatting was not possible given the residents’ condition:

“She was already trapped in her chair, she wasn’t able to do anything and had to stay in her room. She didn’t even see other residents, so it was horrific. (…) I thought it was horrible for that lady”. (V1)

Besides the wellbeing of residents, wellbeing of close relatives was also mentioned as relevant. For some close relatives, especially the partners, visiting their loved ones was very important for their own mental wellbeing, for example when they were married and used to see each other daily.

#### Respect and recognition

Participants reported that residents living in nursing homes did not receive the respect and recognition they felt they deserved. First, residents and close relatives reported feeling discriminated against because they were obliged to follow stricter rules than people not living in nursing homes. In this context, residents compared their situation during the restrictions with prison time as they were unallowed to leave the premises or their apartment/room. A second reason participants felt they lacked respect and recognition is that they were not involved in the decision making process resulting in the restrictions in nursing homes. They reported that it felt like nursing home residents were forgotten by society, written off or clearly not of value to society anymore.

“[And for you, what do you remember most if you think about the first months of the corona period?]

Especially the anger about the fact that no one really listened to the elderly. On the subject of relaxations of the rules, arguments were related to the economic needs. But on the issue of the elderly there was nothing. That was just a forgotten group”.(L11).

#### Hope

Another value reported was (keeping up) hope that better times would come. Participants reported that they missed perspective regarding when the visitor ban would (ever) end. They mentioned that the insecurity about when and how it would end made it difficult.

“Then I think: if you know that it’s only for two weeks, you can say to her: only two weeks without visits, we will call and skype every day, and in two weeks we’re allowed back in to visit you. That’s also unusual, but now we didn’t even know how long it would last. And it lasted and lasted, that was horrible”. (GT1)

### Moral lessons and recommendations

In the interviews, the focus group with volunteers and the Socratic dialogue meetings, we asked the participants what they considered (moral) lessons and what should be taken into account in case of future pandemics. Participants varied in their positions and discussed the pros and cons of the various lessons they deemed important. Below we describe three normative lessons that the participants considered as most important.

#### Tailored (well-balanced) solutions in context

Participants considered both safety as well as social and physical contact between residents and close relatives/volunteers as important for the wellbeing of residents. They advised that policies in crisis should be able to fine-tune restrictions in case the residents’ (mental) wellbeing is on the line. That is, if a resident clearly suffered from the restrictions, it should be possible to make exceptions regarding the restrictions to meet the individual needs and wishes of residents and secure human dignity. In general, participants advised that at least one close relative should be able to visit the resident, and in case there is no close relative, a volunteer should take this role.

“Of course there are all sorts of nursing homes and every situation is different, but I would think: let at least one dedicated person in. So there always is someone with whom the resident feels comfortable with. That’s always different than contact with staff. They’re all excellent, but being with someone you know and love is always better”.(T8).

On the other side, allowing at least one close relative or volunteer in the nursing home per resident was also experienced as a moral dilemma in the Socratic dialogue meetings as it could jeopardize safety. Participants discussed that not all families might take safety measures seriously enough, which could negatively affect the whole group.

“That is actually a problem. There are many people who are very careful, but there are also people who do not believe in the safety policies. They do not want to wear masks or wash hands, because they think it is bullshit”. (SD2:R1)

Fine-tuning restrictions in concrete, urgent cases was considered the regarding of all values and perspectives, including the suffering of the people involved, and together developing a well-balanced strategy.

#### Proper recognition

A second normative lesson was that close relatives, who are closely involved in the care of the resident, should not be identified as a ‘visitor’. Participants reckoned that family carers deserve proper recognition for what they do. The status of ‘visitor’ did not do justice to the role of some close relatives and volunteers who normally performed many caring tasks for residents and were present daily in the nursing homes. One of the husbands explained that suddenly he was no longer considered as a partner in care:

“I think there’s a big difference between visitors and relatives who are also caretaker. It was all lumped together (…) All of a sudden you’re no longer part of it”. (SD3:R4)

#### Responsive power distribution

In line with the above-mentioned advice, a third normative lesson, according to the participants, is that the nursing homes should be given the authority from the government to make tailored policy choices that address the wishes and specific needs of their residents, close relatives and volunteers. In addition, nursing homes should invest in providing clear information for residents, close relatives and volunteers and facilitating on-going dialogue with them. In the Socratic dialogue meetings, participants expressed that they do not necessarily need a final voice in the matter, but want to be heard and involved in decision-making processes. Most important is that nursing homes inform and stay in contact with them. This implies receiving clear information about what is going on and which policy changes are being made. Part of being taken seriously and receiving proper recognition meant the ability to contact staff and get support to reduce their worries. Having a final voice in the matter as a non-professional was experienced as a moral dilemma as it also could jeopardize the safety of the residents. Participants reckoned that the perspectives of residents, close relatives and volunteers can be very diverse, therefore reaching consensus on policies may be difficult. Also, it was considered more appropriate if a professional takes responsibility for final decisions, as they have more expertise on the matter:

“I think you should be a professional as it requires medical knowledge. I believe the discussion is already very complex, so we [family/volunteers] should not complicate it any further” (SD1, R4).

## Discussion

While most participants were nuanced and accepted the restrictions at first, in time, critical perspectives developed concerning the effectiveness of the restrictions, its proportionality, its experienced unfairness and how the restrictions were imposed. The restrictions compromised several values. When considering the importance of sustaining close connections, the participants regarded (physical) safety and the mental wellbeing of residents and close relatives as the most important values. Furthermore, participants reported respect and recognition for residents and close relatives and the role of (keeping up) hope. Three moral lessons were considered by the participants in case a similar situation would arise: (1) it should be possible to construct tailored (well balanced) solutions in practice; (2) close relatives should receive proper recognition for their involvement in care; (3) and power distribution should be responsive and inclusive.

Looking at the moral dynamics from the perspectives of residents, close relatives and volunteers, we notice several patterns that correspond with moral theory. First, the fact that participants at the beginning of the COVID-19 outbreak accepted the restrictions resonates with the moral philosophy of Annette C. Baier [27]. According to Baier, trust in the good of the decisions made by others plays a central role in practices where people are embedded in vulnerable positions and depend on care from others. In case of new situations of dependency and/or insecurity, people involved, in general, will react with compliance (e.g. wait and see, and give the authorities the benefit of the doubt). Especially in a situation where much is still unknown. According to Baier, the given trust must prove itself over time. If not, distrust will grow and people will develop more critical standpoints and frustration. This moral process relates to the experiences of the participants. While people initially accepted the restrictions, in time moral questions developed as moral values were compromised.

Second, that the moral epistemology of the philosopher Margaret Urban Walker can explain critical perspectives developed over time [28]. Her moral theory is based on the practice of morality in real life. Ethics is considered a collaborative social practice, meaning that moral knowledge is constructed and developed in interactions between people. People learn from and through each other what is morally important (e.g., what is worth caring for) and for what reasons. This does not imply that what is considered morally right or good is always straightforward or that people would automatically agree. Different social backgrounds and responsibilities steer different moral stances and can stumble on moral ambiguity or oppositions. Morality, understood as interpersonal knowledge, is constantly under construction [29]. Critical perspectives can be considered as tools that initiate openings to find out if current practices are also the right practices that is if a specific practice is mutually experienced as the best way to live with others within those particular circumstances. The critical perspectives of the participants have been beneficial as new angles for dialogue and negotiation about which policies should prevail. Expressing and acknowledging those values experienced as being challenged in dealing with the COVID-19 outbreak in nursing homes fosters the debate about which moral lessons are important. According to Walker, inclusion of all stakeholders and transparency are essential requirements to realize better moral practices.

For future policies on pandemic outbreaks, we recommend that the moral lessons from the perspective of residents, close relatives and volunteers are included. This is in line with the Convention on the Rights of Persons with Disabilities (CRPD) that proscribes that no policy should be decided without involvement of all stakeholder perspectives that are affected by that policy [30]. Moral case deliberation can be useful regarding the first moral lesson – *construct tailored well-balanced solutions* [31]. This structured dialogue method that addresses moral dilemmas in concrete contexts involving all stakeholder perspectives whose lives are impacted by the concrete dilemma of focus. Regarding the second moral lesson of the participants, *provide proper recognition*, it could be worth looking into what might be needed to construct moral repair [32]. More attention could be paid to the ‘counter-stories’ of residents, close relatives and volunteers on how they experienced the COVID-19 restrictions to steer the dialogue about what is, and who provides, necessary care. Using counter stories is a way to restore the identity of a person or stakeholder group that has been suppressed or ignored in moral practices [33]. The outcomes of the larger research project have been translated into a detailed illustration depicting the various lived experiences of participants during the restriction [17]. This cartoon is used in nursing homes to stimulate ongoing dialogue and stakeholder reflection with stakeholders about the pandemic. Regarding the third lesson, *responsive power distribution*, we recommend sustaining trustful relationships between the nursing homes and close relatives. In case of new in-person ban, providing proactive and detailed information about the situation in the nursing homes and the wellbeing of residents can be helpful. Being well-informed prevents residents and close relatives from becoming angry and distrustful as does being mindful and giving due recognition to those affected by the restrictions [16].

### Strengths & limitations

By involving participants from different (cross-border) regions, we were able to construct a broad outlook of the moral attitudes of nursing home residents, close relatives and volunteers. Yet, as moral dynamics are constructed in social practices which can be influenced and steered by specific cultural backgrounds, a limitation of this study is that moral lessons learned may not be recognized or acknowledged by all representatives of the stakeholder perspectives. For example the gender of the participants was not fully balanced as more participants were female. Morality is always evolving, therefore the lessons are not static or applicable to all residents, close relatives and volunteers in nursing homes. The lessons are not fixed end-points -they are topics that deserve consideration in the moral debate.

Another limitation of this study is that residents with severe dementia were not included in this study because their cognitive impairment hinders verbal expressions of how they experienced the restrictions. In the data collection, close relatives and volunteers expressed how they considered their loved one with severe dementia had experienced the restrictions. These pictures were impaired by the physical distance and the cognitive impairment itself, making it difficult to understand the emotional experiences of this group of residents.

## Conclusions

This study reported on nursing home residents’, close relatives’ and volunteers’ retrospective views on the nursing home COVID-19 restrictions. The analysis revealed three moral lessons or recommendations for consideration when addressing policies for future pandemics: tailored (well-balanced) solutions in context, proper recognition, and responsive power distribution (‘nothing about us, without us’). These formulations are relevant due to their specific attention to the perspectives of stakeholders who were not involved in decision-making even though the restrictions impacted their lives. Addressing these perspectives can contribute to the ongoing debate on creating morally good nursing home practices to deal with future pandemics.

### Supplementary Information

**Additional file 1.**

## Data Availability

Transcripts and analysis are stored at TRANZO, Tilburg School of Social and Behavioral Sciences, Tilburg University. Anonymised data can be requested.
