# Supplementary material for: Moral lessons from residents, close relatives and volunteers about the COVID-19 restrictions in Dutch and Flemish nursing homes
Source: Philos Ethics Humanit Med. 2023 Sep 6;18:12. doi: 10.1186/s13010-023-00140-w (PMC10481572; doi:10.1186/s13010-023-00140-w)
Supplement: Supplementary file 1 — Additional file 1. [file 13010_2023_140_MOESM1_ESM.docx]

**Appendix 1. Semi-structured interview guide interviews with residents and close relatives**

| **Topics** | **Probing questions** |
| --- | --- |
| *General experiences of visitors ban* | How did you experience the visitors ban?  How did the visitors ban affect you?  How did you deal with the visitors ban? What was helpful for you? |
| *Daily activities* | Could you tell me how you usually spend your days?  How has this changed during the visitors ban?  How did that make you feel? How did you deal with this? What was helpful for you? |
| *Social contacts and social needs* | Could you tell me about your social contacts?  What do you think about your social contacts?  What were your social contacts like during the visitors ban?  How did that make you feel? What was helpful for you? |
| *Loneliness* | Do you have a close or intimate relationship with someone?  Do you have as many social contacts as you would want to have?  To what extent can you still do what is important to you?  Have you felt lonely or sad during the visitors ban?  At the moment, do you still experience these emotions? |
| *Other (positive) consequences of restrictive measures and relaxation* | Are there other things that you have experienced or that you think are important to mention about the visitors ban?  What were the positive aspects of not being allowed to receive visitors?  What do you think of the other measures that you and others had to deal with? Are there any aspects that you find positive?  Are there any changes that you would like to keep, for yourself or others?  Could you mention anything that you would have done differently or things that should change? |

**Appendix 2 Semi-structured interview guide focus group with volunteers**

| **Topics** | **Probing questions** |
| --- | --- |
| *Changes in volunteer work during visitors ban* | Were there any changes for you as a volunteer? Could you continue your work as a volunteer?  In case volunteers could not continue their volunteer work: Were you able to keep in touch with residents? |
| *General experiences of visitors ban* | How did you experience the visitors ban?  How did the visitors ban affect you? |
| *Loneliness* | Have you felt lonely or sad during the visitors ban?  How did you deal with this? What was helpful for you?  Have you experienced loneliness or sadness among residents?  How did you deal with this? |
| *Evaluation of visitors ban* | Do you feel the visitors ban was justified?  Could you mention anything that you would have done differently or things that should change?  Do you have any advise or lessons learned for the future? |

**Appendix 3 Protocol Socratic dialogues with residents, close relatives and volunteers (mixed)**

| **Topics** | **Probing questions** |
| --- | --- |
| *VALUES* | *In retrospect, what concerns you the most and should be addressed in case of a new pandemic outbreak in relation to loneliness, social needs and social relations?*  What touched you the most? Why? Which values did you experience? When did you experience this? What happened?  Did you experience moral dilemmas/ contradictions in your attitudes? Which values were on the line? |
| *NORMS* | *What do you think – with the current knowledge about COVID-19 and pandemics – should become minimal standards policies should address?*  What should not be forgotten in case a new pandemic would develop? What are minimal criteria that should be met? What kind of actions/ rules should follow from the values you addressed?  What are borderlines in case values conflict? What should be done next time?  Which downsides would be (morally) acceptable? How could possible downsides be prevented? |
